# Genetic Architecture of the Variation in Male-Specific Ossified Processes on the Anal Fins of Japanese Medaka

**DOI:** 10.1534/g3.115.021956

**Published:** 2015-10-26

**Authors:** Maiko Kawajiri, Shingo Fujimoto, Kohta Yoshida, Kazunori Yamahira, Jun Kitano

**Affiliations:** * Division of Ecological Genetics, National Institute of Genetics, Mishima, Shizuoka 411-8540, Japan; † Tropical Biosphere Research Center and Graduate School of Engineering and Science, University of the Ryukyus, Nishihara, Okinawa 903-0213, Japan; ‡ School of Life Sciences, SOKENDAI, Mishima, Shizuoka 411-8540, Japan

**Keywords:** medaka, median fin, courtship, ornament, genetic correlation

## Abstract

Traits involved in reproduction evolve rapidly and show great diversity among closely related species. However, the genetic mechanisms that underlie the diversification of courtship traits are mostly unknown. Japanese medaka fishes (*Oryzias latipes*) use anal fins to attract females and to grasp females during courtship; the males have longer anal fins with male-specific ossified papillary processes on the fin rays. However, anal fin morphology varies between populations: the southern populations tend to have longer anal fins and more processes than the northern populations. In the present study, we conducted quantitative trait locus (QTL) mapping to investigate the genetic architecture underlying the variation in the number of papillary processes of Japanese medaka fish and compared the QTL with previously identified QTL controlling anal fin length. First, we found that only a few QTL were shared between anal fin length and papillary process number. Second, we found that the numbers of papillary processes on different fin rays often were controlled by different QTL. Finally, we produced another independent cross and found that some QTL were repeatable between the two crosses, whereas others were specific to only one cross. These results suggest that variation in the number of papillary processes is polygenic and controlled by QTL that are distinct from those controlling anal fin length. Thus, different courtship traits in Japanese medaka share a small number of QTL and have the potential for independent evolution.

Traits involved in reproduction evolve rapidly and show great diversity among closely related species (Darwin 1871; Andersson 1994; Mendelson and Shaw 2005; Arnegard *et al.* 2010). For example, modification of a particular body part for reproduction occurs across the animal kingdom, and the presence/absence and the magnitude of the modification can vary among related species (Darwin 1871). Fish median fins are good examples of diverse modifications of external morphology for reproduction. In the males of several fishes, particular rays of the median fins are modified for attracting females and/or copulation during courtship (Meyer 1997; Zauner *et al.* 2003; Kwan *et al.* 2013).

For a particular body part to be modified, that part should be genetically and developmentally decoupled from other body parts because the presence of strong genetic correlations would lead to correlated evolution of other parts (Lande 1979), which is potentially deleterious (Badyaev 2004). Therefore, investigating the genetic architectures underlying variation in courtship traits is indispensable to obtain a better understanding of the evolutionary mechanisms underlying the diversification of courtship traits. Genetic mapping of courtship traits has been extensively conducted in insects, and these studies have demonstrated that quantitative trait loci (QTL) controlling different components of courtship traits occasionally, but not always, overlap (Howard and Berlocher 1998; Orr 2001; Williams *et al.* 2001; Coyne and Orr 2004; Hutten *et al.* 2004; Yeh and True 2014). In contrast, little is known about the genetic architecture underlying the variation in courtship traits in vertebrates (Kitano *et al.* 2009; Schielzeth *et al.* 2012; Santos *et al.* 2014).

Medaka fishes of genus *Oryzias* provide a good system for investigations into the genetic architecture underlying courtship traits because sexually dimorphic traits, including modified median fins, exhibit variation between closely related species (Iwamatsu 2006). In *Oryzias*, males generally have longer anal and dorsal fins than females, although the magnitude of sexual dimorphism differs between species and even populations (Iwamatsu 2006; Kawajiri *et al.* 2009, 2014). For example, southern Japanese medaka (*Oryzias latipes*) males have longer anal and dorsal fins than northern males (Kawajiri *et al.* 2009; Kawajiri and Yamahira 2011). Experiments examining Japanese medaka indicate that longer anal fins are likely to increase reproductive success in males. During courtship of Japanese medaka, the male and the female are aligned side-by-side in the same direction, and the male wraps the female with his dorsal and anal fins; then, the female spawns her eggs and the male ejaculates his sperm. Previous research indicated that experimental shortening of male anal fins reduced the fertilization rate (Egami and Nambu 1961; Koseki *et al.* 2000). Additionally, a behavioral study showed that females preferred males with longer fins (Fujimoto *et al.* 2014).

Males of several medaka species also possess male-specific ossified processes called papillary processes (Figure 1); in Japanese medaka, the expression of these processes is induced by androgens (Ogino *et al.* 2013; Nakamura *et al.* 2014). The presence and the number of papillary processes differ between species and populations. For example, all Japanese medaka males possess papillary processes; however, medaka species from Indonesia lack papillary processes, although Indonesian males have longer anal fins than females. Papillary processes are thought to rub the female to induce spawning (Ono and Uematsu 1957), although experimental tests of the functions of papillary processes have not yet been conducted. Furthermore, Japanese medaka females sometimes reject the release of eggs by disentangling themselves from the male’s anal fins (Fujimoto *et al.* 2015), and the papillary processes in the males may prevent the females from escaping. Thus, the length of the anal fin and the presence of papillary processes show different patterns of variation in the genus *Oryzias*.

**Figure 1:**
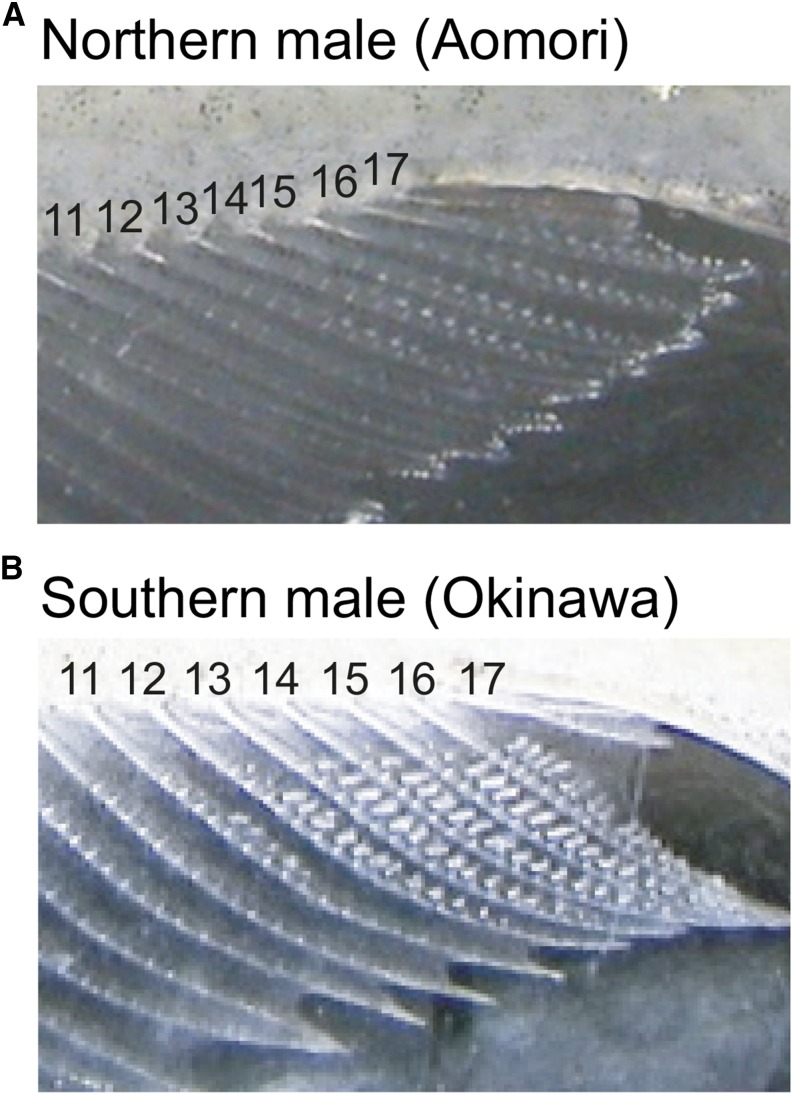
Representative photos of the posterior parts of the anal fins of a northern male (A) and a southern male (B). The numbers in the figures indicate the fin rays analyzed in this study.

The present study had two main aims. First, we conducted QTL mapping of the number of papillary processes on the anal fin in an F_2_ family that was used previously for QTL mapping of the anal fin length (F_2_ intercross derived from a southern Japanese medaka female and a northern Japanese medaka male) (Kawajiri *et al.* 2014). This result was used to test whether the QTL for the papillary process numbers overlapped with the previously identified QTL for the anal fin length. Second, we made another independent cross derived from a northern Japanese medaka female and a southern Japanese medaka male and conducted QTL mapping of both the papillary process numbers and the anal fin length. This result was used to test the number of QTL that were replicated between two independent F_2_ crosses. Because multiple alleles with different effects are likely segregating within natural populations (Mauricio 2001), QTL mapping with multiple crosses is necessary to understand the genetic variation underlying phenotypic variation in natural populations (Barnwell and Noor 2008).

## Materials and Methods

### Fish collection and crosses

Pictures of wild-caught adult medaka fishes that were taken previously were used for the analysis of wild-caught fishes. The southern population was collected from Ginama, Okinawa Prefecture, Japan (26°83**′**N, 128°26**′**E) in 2012, whereas the northern population was collected from Mayajiri, Aomori Prefecture, Japan (40°50**′**N, 140°49**′**E) in 2006 and 2011. In the laboratory, 22 southern males and 20 northern males were photographed individually from the side with the use of a Pentax Optio W90 (Ricoh Imaging, Tokyo, Japan) and Coolpix 4500 (Nikon, Tokyo, Japan), respectively. The papillary processes on each anal fin ray of each male were counted with the photographs.

To make F_2_ crosses, the same populations were collected in 2011. One F_2_ cross was performed with an Okinawa female and an Aomori male (termed OFAM) that had been used for QTL mapping of the anal fin length, as reported elsewhere (Kawajiri *et al.* 2014). This family consisted of 78 males and 92 females. We performed another F_2_ cross with an Aomori female and an Okinawa male (termed AFOM). This new cross was performed according to previously described procedures (Kawajiri *et al.* 2014). To summarize, one female from the north and one male from the south were crossed to create F_1_ progeny. Next, F_1_ females and F_1_ males were allowed to mate to produce an F_2_ family composed of 102 males and 87 females. The F_2_ progeny were grown from the egg stage individually in single acrylic containers (50 × 100 mm with a depth of 200 mm) and maintained under a 14:10-hr light-dark cycle.

### Morphological analysis of the F_2_ crosses

Each fish was photographed at 11 ages: 15, 20, 27, 34, 43, 52, 63, 74, 89, 104, and 124 d after fertilization (DAF). For photographing, the fish were placed in a narrow transparent acrylic box and photographed from the side with a digital camera (Pentax Optio W90, Ricoh Imaging, Tokyo, Japan) as described previously (Kawajiri *et al.* 2009). The papillary processes on each anal fin ray were counted with only the photographs taken at DAF 124, because fish at DAF 104 and younger were too small to allow counting of the papillary processes based on the pictures.

The standard length and anal fin length of each fish were measured at each age to the nearest 0.01 mm with Adobe Illustrator 10.0.3 (Adobe Systems, San Jose, CA) with the plug-in software BPT-Pro2 (Baby Universe, Fujisawa, Kanagawa, Japan). The anal fin length was measured at the fourth anal fin ray, as described previously (Kawajiri *et al.* 2009, 2014).

In addition to the fin length at each age, we measured the growth curve shapes of the anal fins using orthogonal polynomial regression. Details regarding the methods were described previously (Kawajiri *et al.* 2014), and the R scripts used are available from Dryad (http://dx.doi.org/10.5061/dryad.nq36d). In brief, a cubic spline was fitted to the mean of the growth trajectories of all F_2_ fish, and the residuals from the mean were calculated for each F_2_ individual. Third-order Legendre polynomials were fitted to these residuals. Then, we derived the weights of orthogonal polynomials from each fit to describe the curve shape of each F_2_ fish; these weights (from order 0 through order 3) were used for QTL mapping.

### Linkage map construction and QTL mapping

The linkage map of the OFAM family was previously reported (Kawajiri *et al.* 2014). The linkage map of the AFOM family was constructed according to previously described procedures (Kawajiri *et al.* 2014). In brief, genomic DNA was isolated from the right pectoral fin and the right eye of each F_2_ fish with a DNeasy Blood &Tissue Kit (QIAGEN, Valencia, CA). Single-nucleotide polymorphism (SNP) genotyping of the grandparents and F_2_ progeny was conducted with the custom VeraCode GoldenGate Genotyping Assay with 384 SNP panels (Illumina, San Diego, CA) that were previously designed for Japanese medaka fish (Kawajiri *et al.* 2014). Details regarding the design of the SNP panel are available from Dryad (http://dx.doi.org/10.5061/dryad.nq36d). The data were analyzed with Illumina GenomeStudio software, and 273 successfully genotyped SNPs were used for linkage map construction. JoinMap 3.0 software was used to construct a linkage map with a LOD threshold of 4.0 (Van Ooijen and Voorrips 2001).

QTL mapping of the papillary process number was conducted by multiple QTL mapping in R/qtl (Broman and Sen 2009; Arends *et al.* 2010). Unsupervised backward elimination was used to find cofactors. Genome-wide permutation tests (1000 bootstraps) were conducted to find significant QTL (*P* < 0.05). Significant QTL were used to construct a final model, and the calculation of the percentage of variance explained by each QTL and *F*-tests of the effects of QTL were conducted with the fitqtl function in R/qtl. Because multiple QTL mapping in R/qtl cannot be used to analyze X chromosomes, we excluded the X chromosome (LG1) from the analysis. Instead, we conducted a genome scan with a single QTL model (the function scanone in R/qtl), but we did not find any significant QTL on LG1. Therefore, we determined that the exclusion of LG1 did not affect our conclusions. The dominance of each QTL was analyzed with the function effectscan in R/qtl. A |*d*/*a*| ratio > 0.5 was considered dominant, while a |*d*/*a*| ratio < 0.5 was considered additive (no QTL had a |*d*/*a*| ratio > 1.0) (Bernatchez *et al.* 2010). The genotype and phenotype data that can be used to reproduce the results are available from Dryad (doi: 10.5061/dryad.5v71p).

### Statistical analysis

A Mann-Whitney *U*-test was conducted to compare the number of papillary processes between populations using the exactRankTests R package (Hothorn and Hornik 2015). Pearson’s correlation coefficients were calculated to test the correlation between traits. *P*-values of LOD scores from the QTL mapping were determined with 1000 genome-wide bootstraps. When the 95% Bayesian credible intervals overlapped, two QTL were considered to overlap.

### Data availability

The genotype and phenotype data are available from Dryad (doi: 10.5061/dryad.5v71p). R scripts for orthogonal polynomial regression are available from Dryad (http://dx.doi.org/10.5061/dryad.nq36d).

## Results

### Variation in the number of papillary processes

**Figure 2:**
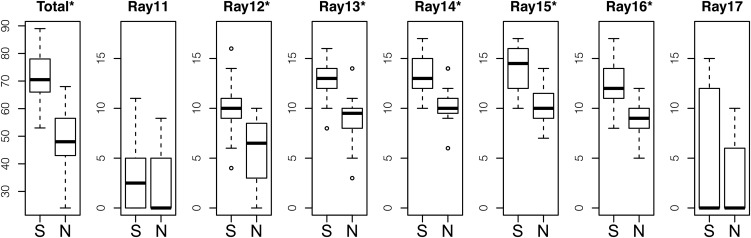
Boxplots of the number of papillary processes. The total number of papillary processes (Total) and the number of papillary process on each fin ray (Ray) were compared between the southern males (S) and the northern males (N). The asterisks indicate significant differences in the traits between crosses by the Mann-Whitney *U*-test (*P* < 0.001). N = 22 for the southern population and N = 20 for the northern population. Because only two southern males had papillary processes on Ray 10 and only one northern male had a papillary process on Ray 18, these two fin rays were not included in the analysis.

We first compared the number of papillary processes of wild-caught fish between populations (Figure 2). The southern males had a significantly greater total number of papillary processes than the northern males (Mann-Whitney *U*-test, W = 414, *P* < 0.001). Then, we examined the number of papillary processes on each fin ray separately. We found that the number of papillary processes on Rays 12−16 significantly differed between populations, with the southern males having more processes than the northern males (Mann-Whitney *U*-test, *P* < 0.001).

### Genetic mapping of the variation in the number of papillary processes in the OFAM family

**Figure 3:**
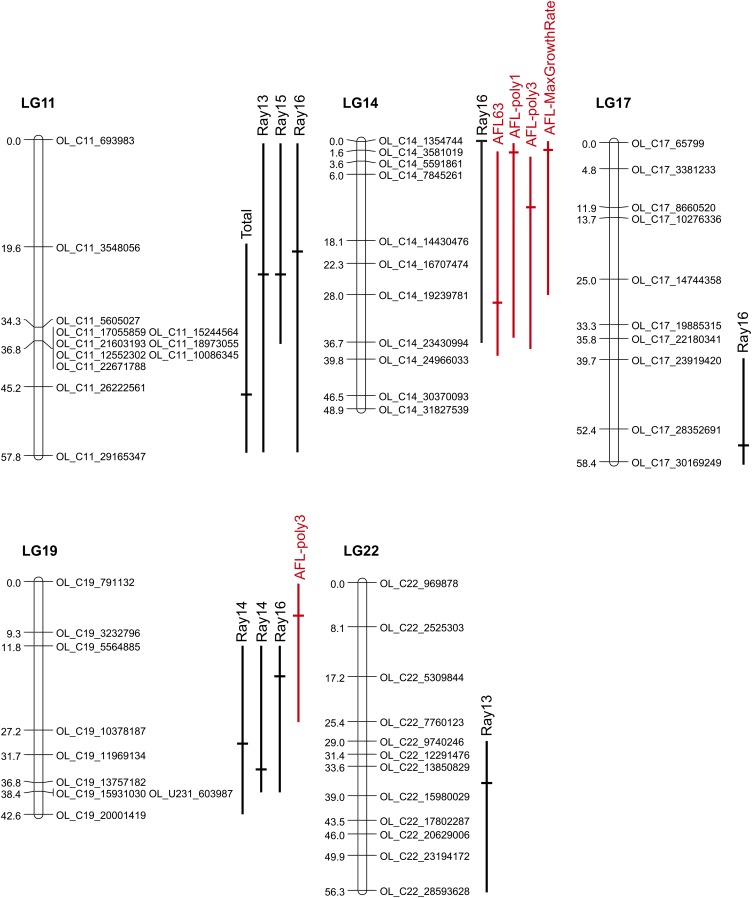
Significant quantitative trait loci (QTL) and 95% Bayesian credible intervals mapped on the linkage groups (LGs) of the OFAM family. Only LGs with significant QTL are shown here. Black and red lines indicate QTL for papillary processes and anal fin length, respectively.

**Figure 4:**
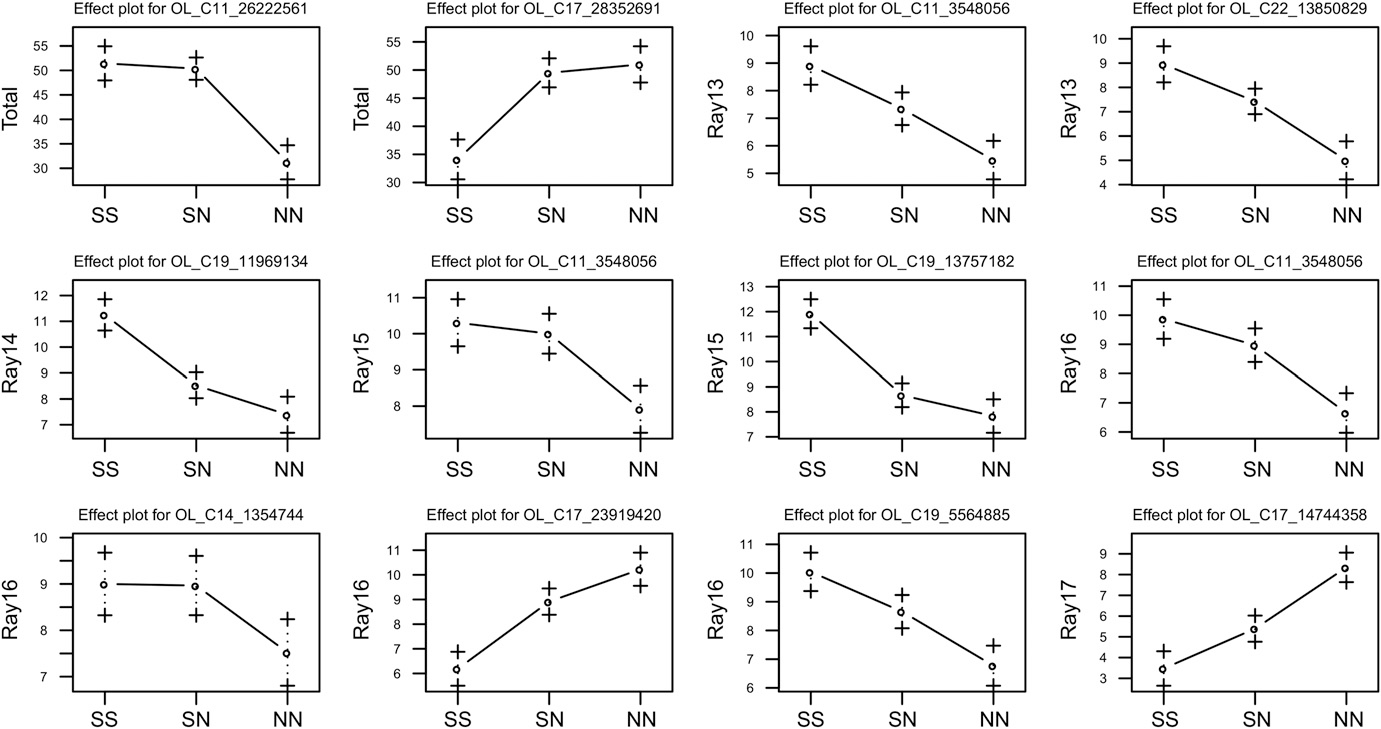
Comparison of the number of papillary process among genotypes at the single-nucleotide polymorphism marker nearest the QTL in the OFAM family: SS, homozygotes of the southern alleles; SN, heterozygotes; NN, homozygotes of the northern alleles.

QTL mapping of the total number of papillary processes in the OFAM family revealed two significant QTL (Table 1, Figure 3, and Supporting Information, Figure S1). A comparison of phenotypic traits among three genotypes (*i.e.*, southern homozygotes, heterozygotes, and northern homozygotes) at these loci showed that the QTL on LG11 had an expected direction of effect such that the southern allele increased the total number of papillary processes (Figure 4). In contrast, the QTL on LG17 had the opposite direction of effect: the southern allele reduced the total number of papillary processes (Figure 4). The southern allele was dominant at the QTL on LG11 (|*d*/*a*| = 0.892), whereas the northern allele was dominant at the QTL on LG17 (Figure 4, Table 1; (|*d*/*a*| = 0.822)).

**Table 1:** QTL controlling the number of papillary processes in the OFAM family

Trait	LG	Location, cM	95% BI, cM	Nearest Maker	LOD*^a^*	Threshold*^b^*	PVE	*P*-value*^c^*	Additive Effect (*a*)*^d^*	Dominance Effect (*d*)	Dominance (|*d*/*a*|)
Total	11	45	19.6−57.8	OL_C11_26222561	4.09	3.99	18.4	< 0.001	10.11	9.02	0.892*
Total	17	55	39.7−58.4	OL_C17_28352691	4.43	3.99	11.2	0.003	−8.45	6.95	0.822*
Ray13	11	25	0−57.8	OL_C11_3548056	3.12	3.10	14.0	0.001	1.71	0.15	0.086
Ray13	22	35	29.0−56.3	OL_C22_13850829	3.14	3.10	14.6	0.001	1.98	0.45	0.228
Ray14	19	30	11.8−42.6	OL_C19_11969134	5.52	3.99	21.4	< 0.001	1.93	−0.79	0.410
Ray15	11	25	0−36.8	OL_C11_3548056	3.29	3.20	8.2	0.016	1.20	0.89	0.745*
Ray15	19	35	11.8−38.4	OL_C19_13757182	8.67	3.20	21.1	< 0.001	2.04	−1.20	0.592*
Ray16	11	20	0−57.8	OL_C11_3548056	3.43	2.91	6.3	0.018	1.61	0.71	0.440
Ray16	14	0	0−36.8	OL_C14_1354744	3.23	2.91	5.3	0.033	0.74	0.70	0.953*
Ray16	17	45	25.0−52.4	OL_C17_23919420	9.05	2.91	24.0	< 0.001	−2.02	0.71	0.349
Ray16	19	15	11.8−38.4	OL_C19_5564885	4.10	2.91	8.6	0.005	1.63	0.25	0.152
Ray17	17	25	13.7−52.4	OL_C17_14744358	3.87	2.60	21.3	< 0.001	−2.44	−0.52	0.211

Asterisks indicate QTL with dominant effects (dominancy > 0.5). QTL, quantitative trait loci; LG, linkage group; BI, Bayesian credible intervals; PVE, percentage of variance explained; MQM, multiple QTL mapping.

a Peak LOD scores were calculated by MQM analysis.

b LOD thresholds of *P* < 0.05 were calculated by genome-wide permutation tests.

c *P*-values of the effects of genotypes were calculated by the *F*-test of the fitqtl function.

d Positive values of additive effects indicate that the southern alleles increase the trait values.

Next, we conducted QTL mapping of the number of papillary processes on each fin ray separately. We found at least one significant QTL for the number of papillary processes on Rays 13−17, whereas no significant QTL were found for Ray 11 or Ray 12 (Table 1, Figure 3, and Figure S1). In addition to LG11 and LG17, we found significant QTL on LG14, LG19, and LG22, suggesting that these QTL were specific to particular fin rays and contribute little to the total number of papillary processes. A comparison of phenotypic traits among genotypes of these fin ray−specific QTL showed that all of these QTL on LG14, LG19, and LG22 had the expected directions of effects (Figure 4). However, the degree of dominance varied among QTL with |*d*/*a*| ranging from 0.152 to 0.953 (Table 1). A significant interaction was only found between LG11 (24 cM) and LG19 (34 cM) for Ray 15 (*P*-value of *F*-test of the interaction = 0.0249, percentage of variance explained by interaction = 9.6): the QTL on LG11 became effective in the presence of the northern allele at LG19 (Figure S2).

**Figure 5:**
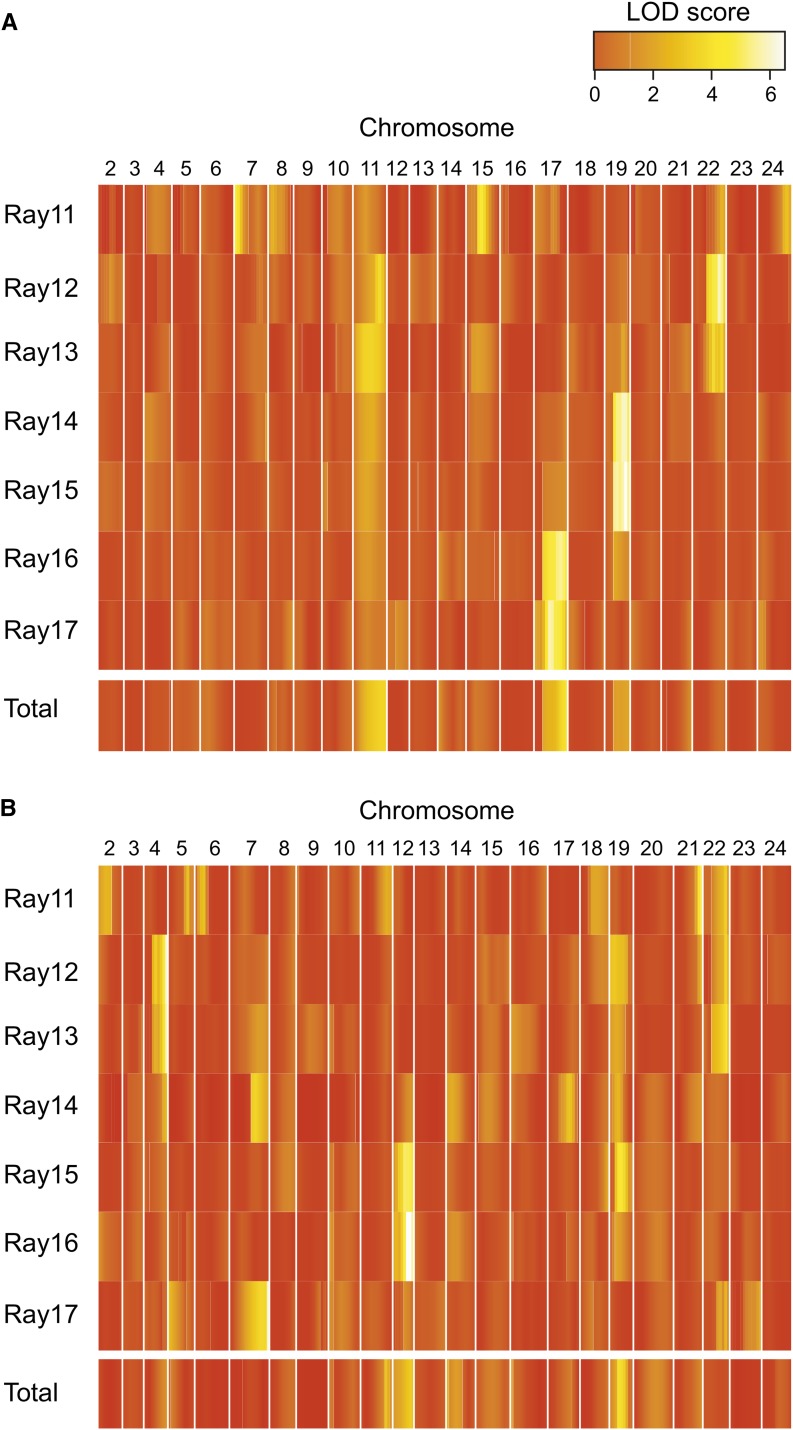
Heat maps of the LOD scores for each fin ray and the total number of papillary processes are shown for the OFAM (A) and AFOM families (B).

A heat map of the LOD scores revealed that different fin rays were controlled by different QTL, although nearby fin rays tended to share QTL (Figure 5). For example, the profiles of the LOD scores appeared to be similar either between Ray 12 and Ray 13, between Ray 14 and Ray 15, or between Ray 16 and Ray 17. However, the profiles of the LOD scores of Rays 12-13 appeared to differ compared to those of Rays 16−17 (see also Figure S1). These results indicate that the numbers of papillary processes on different parts of the anal fins are controlled by different QTL.

Because the number of papillary processes was correlated with the standard length and anal fin length (Table S1), we conducted QTL mapping with either the standard length or anal fin length as a covariate. The detected QTL were similar to those detected without covariates (Table S2 and Table S3), suggesting that these QTL were controlling the numbers of papillary processes even after correction for the standard length or the anal fin length.

### Tests of QTL repeatability in the AFOM family

**Figure 6:**
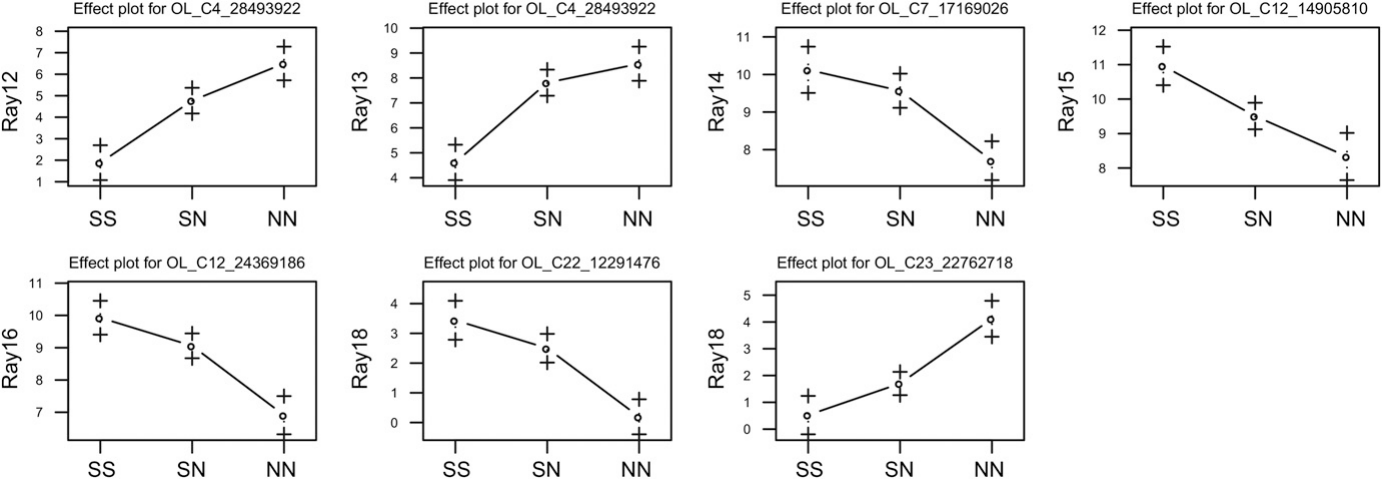
Comparison of the number of papillary processes among genotypes at the single-nucleotide polymorphism marker nearest the QTL in the AFOM family: SS, homozygotes of the southern alleles; SN, heterozygotes; NN, homozygotes of the northern alleles.

To test the QTL repeatability, we conducted QTL mapping on an independent F_2_ family: the AFOM family. We found significant QTL on LG4, LG7, LG12, LG22, and LG23 (Figure S3), which are summarized in Table 2. All QTL except those on LG4 and LG23 had the expected directions of effects (*i.e.*, the southern alleles increased the number of papillary processes) (Figure 6).

**Table 2:** QTL controlling the number of papillary processes in the AFOM family

Trait	LG	Location, cM	95% BI, cM	Nearest Maker	LOD*^a^*	Threshold*^b^*	PVE	*P*-value*^c^*	Additive effect (*a*)*^d^*	Dominance effect (*d*)	Dominance (|*d*/*a*|)
Ray12	4	40	34.7−42.1	OL_C4_28493922	4.50	2.86	15.4	< 0.001	−2.30	0.58	0.25
Ray13	4	40	34.7−42.1	OL_C4_28493922	3.94	3.37	15.8	< 0.001	−1.97	1.22	0.62*
Ray14	7	45	37.3−68.0	OL_C7_17169026	2.43	2.35	10.2	0.005	1.21	0.65	0.54*
Ray15	12	20	14.5−39.7	OL_C12_14905810	3.56	3.03	7.9	0.015	1.31	−0.14	0.11
Ray16	12	30	14.5−39.6	OL_C12_24369186	2.61	2.38	13.0	< 0.001	1.51	0.64	0.42
Ray18	22	30	0−35.8	OL_C22_12291476	3.66	3.06	11.9	< 0.001	1.62	0.68	0.42
Ray18	23	58	25−7-58.0	OL_C23_22762718	3.18	3.06	11.4	0.001	−1.80	−0.62	0.35

Asterisks indicate QTL with dominant effects (dominancy > 0.5). LG, linkage group; BI, Bayesian credible intervals; PVE, percentage of variance explained; MQM, multiple QTL mapping.

a Peak LOD scores were calculated by MQM analysis.

b LOD thresholds of *P* < 0.05 were calculated by genome-wide permutation tests.

c *P*-values of the effects of genotypes were calculated by the *F*-test of the fitqtl function.

d Positive values of additive effects indicate that the southern alleles increase the trait values.

Only one QTL on LG22 overlapped with the QTL found in the OFAM family. A comparison of the heat maps of the LOD scores between the OFAM and AFOM families showed that few QTL were shared between families (Figure 5). When we reduced the LOD significance threshold from *P* = 0.05 to *P* = 0.1, several suggestive QTL were found (Table S4): the QTL on LG22 (40 cM) for Ray 12, the QTL on LG22 (40 cM) for Ray 13, the QTL on LG7 (45 cM) for Ray 14, and the QTL on LG19 (20 cM) for Ray 15. Therefore, in addition to the QTL on LG22, the QTL on LG19 may also be shared between the two families.

Next, we tested whether the shared QTL on LG19 and LG22 had similar effects between families. The QTL on LG22 had a similar effect: in both families, it had no dominance effects (|d/a| < 0.5), and the southern allele increased the number of papillary processes (Table 1 and Table 2). In contrast, the QTL on LG19 had different directions of effects: it had the opposite direction of effect in AFOM (Table S4), whereas it had the expected direction of effect in OFAM (Table 1).

In the AFOM family, we also found significant correlations between the papillary process number and the standard length or the anal fin length (Table S5). However, the inclusion of the standard length or the anal fin length as a covariate did not qualitatively change our results (Table S6 and Table S7).

### Little overlap between QTL for papillary process number and anal fin length

As described previously, there are phenotypic correlations between the raw values of the papillary process number and the anal fin length (Figure S4). Because both the papillary process number and the anal fin length were correlated with the standard length, we calculated the residuals of these traits against the standard length. Significant correlations were found even between the residuals after we corrected for the standard length (Figure S5), suggesting that some QTL for the papillary process number and the anal fin length might be shared.

In OFAM, we compared QTL for variations in papillary processes with previously identified QTL for anal fin length (Kawajiri *et al.* 2014). The QTL on LG14 and LG19 overlapped (Figure 3), whereas other QTL on LG11, LG17, and LG22 did not. Overlapping QTL on both LG14 and LG19 had the expected directions of effects on both traits: the southern alleles increased both the anal fin length (Kawajiri *et al.* 2014) and the number of papillary processes (Figure 4).

Because we did not perform QTL mapping of the anal fin length and anal fin growth curves in the AFOM family in our previous study (Kawajiri *et al.* 2014), we conducted QTL mapping of these traits in the AFOM. When the anal fin length was analyzed separately at each age, we found significant QTL on LG10 and LG15 (Table S8; Figure S6). When the weights of the orthogonal polynomial growth curves were analyzed, we found significant QTL on LG13 and LG14 in addition to LG10 and LG15 (Table S8; Figure S6). The QTL on LG14 and LG15 had the expected directions of effects (*i.e.*, the southern alleles increased the anal fin length), whereas the QTL on LG10 and LG13 had the opposite directions of effects (*i.e.*, the northern alleles increased the fin length) (Figure S7). A comparison with QTL found in the OFAM family revealed that the QTL on LG10 and LG14 were shared between the AFOM and OFAM families. The directions of effects of the QTL on LG10 and LG14 were the same between the AFOM and OFAM families. Finally, we found no overlapping QTL between anal fin length/growth curves and papillary processes in the AFOM family.

## Discussion

### Independent genetic control of anal fin length and the number of papillary processes

A major finding in our study was that there was little QTL overlap between anal fin length and the number of papillary processes in Japanese medaka. Thus, the two courtship traits are not necessarily controlled by the same genetic loci. Furthermore, we found that the numbers of papillary processes on different fin rays were controlled by different QTL. These results suggest that the different courtship traits associated with the anal fins can evolve independently. Consistent with this hypothesis, the genus *Oryzias* exhibits diversity in anal fins. Indonesian *Oryzias* species exhibit sexual dimorphism in anal fin length, with males having longer fins than females but lacking papillary processes (Iwamatsu 2006). Furthermore, *Oryzias mekongensis* males have less sexually dimorphic and shorter anal fins, although they still have papillary processes on the anal fins (Iwamatsu 2006). The independent genetic control of each trait may enable the genus *Oryzias* to exhibit sexual dimorphism diversity in anal fin morphology.

The clustering of genes controlling functionally related traits may be favored when two divergent populations are faced with gene flow because physical linkage can prevent functionally related genes from being separated by recombination (Noor *et al.* 2001; Jiggins and Bridle 2004; Yeaman and Whitlock 2011). For example, clusters of genes involved in reproductive isolation are found in linkage maps of sympatric pairs but not allopatric pairs (Noor *et al.* 2001; Brown *et al.* 2004). In the present study, we used allopatric populations near the northern and southern limits of medaka distribution. Unless there was gene flow between the divergent populations, the physical linkage of QTL for different components of courtship would not necessarily be favored (Feder *et al.* 2013).

### Repeatability of QTL

We noted that some QTL were found in two independent crosses whereas others were not. There are several possible reasons why QTL often fail to replicate across different families (Mauricio 2001; Barnwell and Noor 2008). First, grandparents are simply random samples from the parental populations and do not reflect all of the genetic variation existing in the parental populations (Barnwell and Noor 2008). Unlike inbred laboratory animals, many alleles with different effects are likely segregating within wild populations (Mauricio 2001). This may be particularly true when the analyzed traits are polygenic and/or selection is weak. Second, small sample sizes often lead to the failure of detection of significant QTL (Beavis 1994, 1998). Although our sample sizes were relatively small, this factor alone could not explain the failure to replicate QTL because we did not find any suggestive QTL in one cross that overlapped significant QTL in the other cross, even when the significance threshold of the LOD score was reduced. Moreover, a comparison of the heat maps showed that the LOD score profiles apparently differed between the two families. Third, some epistatic interaction or environmental effects may mask the QTL effects (Vikram *et al.* 2011). OFAM is derived from a grandmother from Okinawa and a grandfather from Aomori, while AFOM is derived from a grandmother from Aomori and a grandfather from Okinawa. Therefore, if sex chromosomes (*i.e.*, Y chromosomes) or maternal factors (*i.e.*, mitochondrial DNA) had any epistatic interaction with QTL, we would detect different QTL between the OFAM and AFOM families. Consistent with this idea, Figure S4 indicates that the OFAM males (upper panel) tended to have a relatively smaller number of papillary processes than the AFOM males (lower panel).

Although some QTL were not repeatable, we found several replicated QTL between families. Divergent alleles may segregate at relatively higher frequencies at these loci. It is also possible that the effects of these QTL may be independent of any epistatic or environmental effects.

### Candidate genes

Several genes are known to control fin development or regeneration within 95% Bayesian credible intervals of QTL (Table 3). One QTL for the papillary process number that was replicated in both families contained *bmp4*. Inhibition of *bmp4* has been suggested to disrupt fin growth during zebrafish fin regeneration (Smith *et al.* 2006). One QTL associated with both anal fin length and papillary process number in the OFAM family contained *frem2a* (Carney *et al.* 2010) and *ar* (Ogino *et al.* 2013). The *ar* gene encodes the androgen receptor (Ogino *et al.* 2013) and androgens can induce the expression of papillary processes on the anal fins of Japanese medaka (Ogino *et al.* 2013; Nakamura *et al.* 2014). Finally, a previous report demonstrated that variation in anal fin shape between a Japanese population and a Korean population of *Oryzias latipes* was associated with the genotype at *cyp1b1* (Katsumura *et al.* 2014), and we found a significant QTL for anal fin length in the AFOM family near *cyp1b1* on LG15 (Table S8). These genes would be good starting points for further investigation into the molecular mechanisms underlying the diversification of anal fin processes and length.

**Table 3:** List of candidate genes

Trait	Gene ID	Gene Name*^a^*	LG	Start	End	Gene Description	References
Process	ENSORLG00000016130	*sall4*	7	25177856	25182920	Spalt-like transcription factor 4	(Harvey and Logan 2006)
Process	ENSORLG00000010025	*prdm1b*	11	20033005	20039258	PR domain containing 1b	(Mercader *et al.* 2006)
Process	ENSORLG00000004542	*dlx6a*	11	9805243	9806998	Distal-less homeobox 6a	(Heude *et al.* 2014)
Process	ENSORLG00000004561	*dlx5a*	11	9810402	9811985	Distal-less homeobox 5a	(Heude *et al.* 2014)
Process	ENSORLG00000005050	*evx1*	11	10570435	10572564	Even-skipped homeobox 1	(Schulte *et al.* 2011)
Process	ENSORLG00000003520	*frem2b*	13	7377750	7446363	Fras1 related extracellular matrix protein 2b	(Carney *et al.* 2010)
Process	ENSORLG00000004105	*frem2a*	14	10458932	10523791	Fras1 related extracellular matrix protein 2a	(Carney *et al.* 2010)
Process	ENSORLG00000013304	bmp4*	22	7364080	7371224	bone morphogenetic protein 4	(Murciano *et al.* 2002; Smith *et al.* 2006)
Length	ENSORLG00000006087	*fgf16*	10	13797026	13799816	fibroblast growth factor 16	(Nomura *et al.* 2006)
Length	ENSORLG00000007648	wnt8a*	10	17132768	17134344	wingless-type MMTV integration site family, member	(Azevedo *et al.* 2011)
Length	ENSORLG00000007667	wnt8a*	10	17134906	17136553	wingless-type MMTV integration site family, member	(Azevedo *et al.* 2011)
Length	ENSORLG00000008220	*ar* (1 of 2)	10	18354970	18362159	androgen receptor beta	(Ogino *et al.* 2013)
Length	ENSORLG00000010026	fgf20b*	10	23453265	23454136	fibroblast growth factor 20a	(Whitehead *et al.* 2005)
Length	ENSORLG00000009520	*ar*	14	17226291	17257141	androgen receptor	(Ogino *et al.* 2013)
Length	ENSORLG00000000853	*cyp1b1*	15	2735154	2737995	cytochrome P450, family 1, subfamily B, polypeptide 1	(Katsumura *et al.* 2014)

Genes involved in fin development and/or regeneration within 95% Bayesian credible intervals of QTL controlling the number of papillary process (Process) and the anal fin length (Length) are shown. LG, linkage group; BI, Bayesian credible intervals; QTL, quantitative trait loci.

a Asterisks indicate genes reported to play important roles in fin regeneration rather than development.

However, it should be noted that we only used 384 SNP panels in this study; therefore, we may have overlooked several QTL with small effects. Even if all of the QTL were captured, their precise locations could not be accurately mapped. Increasing the number of markers using next-generation sequencing technologies would help to identify the causative genes in future studies (Baird *et al.* 2008; Elshire *et al.* 2011; Peterson *et al.* 2012; Glazer *et al.* 2015; Kirchmaier *et al.* 2015). Elongation of fins can be achieved by increasing the number of segments and/or elongating each segment (Iovine 2007; Perathoner *et al.* 2014). Elongation of anal fins in the southern population of the Japanese medaka is mainly caused by an increase in the number of segments (Kawajiri and Yamahira 2011); therefore, further molecular developmental studies would also help to identify the causative genes. Due to the presence of phenotypic diversity between closely related species (Iwamatsu 2006; Mokodongan and Yamahira 2015) and the availability of gene manipulation technologies (Kinoshita *et al.* 2009; Ansai *et al.* 2013; Ansai and Kinoshita 2014), the genus *Oryzias* will serve as a good model system for further investigation into the molecular mechanisms underlying natural variation in sexually dimorphic or sex-specific traits between closely related species and populations.

## Supplementary Material

Supporting Information
