# Supplementary material for: Genetic Architecture of the Variation in Male-Specific Ossified Processes on the Anal Fins of Japanese Medaka
Source: G3 (Bethesda). 2015 Oct 26;5(12):2875–84. doi: 10.1534/g3.115.021956 (PMC4683658; doi:10.1534/g3.115.021956)
Supplement: Supporting Information [file supp_5_12_2875__index.html]

Genetic Architecture of the Variation in Male-Specific Ossified Processes on the Anal Fins of Japanese Medaka — Supporting Information 

# Genetic Architecture of the Variation in Male-Specific Ossified Processes on the Anal Fins of Japanese Medaka

## Supporting Information for Kawajiri *et al.*, 2015

**Files in this Data Supplement:**

- Figure S1 - LOD score of the number of total papillary process and the number of papillary process for each fin ray from Ray11 through Ray17 in the OFAM family. (.pdf, 152 KB)
- Figure S2 - Effects of interaction between LG11 (OL\_C11\_3548056) and LG19 (OL\_C19\_13757182) on the papillary process number in the OFAM family. (.pdf, 71 KB)
- Figure S3 - LOD score of the number of total papillary process and the number of papillary process for each fin ray from Ray11 through Ray17 in the AFOM family. (.pdf, 146 KB)
- Figure S4 - Correlations between the anal fin length and the total number of papillary processes in the OFAM (upper panel) and AFOM families (lower panel). (.pdf, 100 KB)
- Figure S5 - Correlations between the residuals of anal fin length regressed against standard length (X-?‐axis) and the residuals of the total number of papillary processes regressed against standard length (Y-?‐axis) in the OFAM (upper panel) and AFOM families (lower panel). (.pdf, 93 KB)
- Figure S6 - Significant QTL and 95% Bayesian credible intervals mapped on the linkage groups (LG) in AFOM. (.pdf, 79 KB)
- Figure S7 - QTL effects on the residuals of fin length in the AFOM shown against standard length; red, homozygote of the southern population alleles; gray, heterozygote; blue, homozygote of the northern population alleles. (.pdf, 1,369 KB)
- Table S1 - Phenotypic correlations between traits in the OFAM family. (pdf, 58 KB)
- Table S2 - QTLs for the number of papillary processes analyzed with standard length as a covariate in the OFAM family. (.pdf, 60 KB)
- Table S3 - QTLs for the number of papillary processes analyzed with anal fin length as a covariate in the OFAM family. (.pdf, 62 KB)
- Table S4 - Suggestive QTLs controlling papillary process number in the AFOM. (.pdf, 62 KB)
- Table S5 - Phenotypic correlations between traits in AFOM. (.pdf, 58 KB)
- Table S6 - QTLs for the number of papillary processes analyzed with standard length as a covariate in the AFOM family. (.pdf, 60 KB)
- Table S7 - QTLs for the number of papillary processes analyzed with standard length as a covariate in the AFOM family. (.pdf, 60 KB)
- Table S8 - QTLs controlling anal fin length and growth in the AFOM family. (.pdf, 63 KB)
- Supporting Information - File contains all figures and tables. (.pdf, 1,736 KB)
