# Supplementary material for: Genetic Architecture of the Variation in Male-Specific Ossified Processes on the Anal Fins of Japanese Medaka
Source: G3 (Bethesda). 2015 Oct 26;5(12):2875–84. doi: 10.1534/g3.115.021956 (PMC4683658; doi:10.1534/g3.115.021956)
Supplement: Supporting Information [file supp_g3.115.021956_FigureS2.pdf]

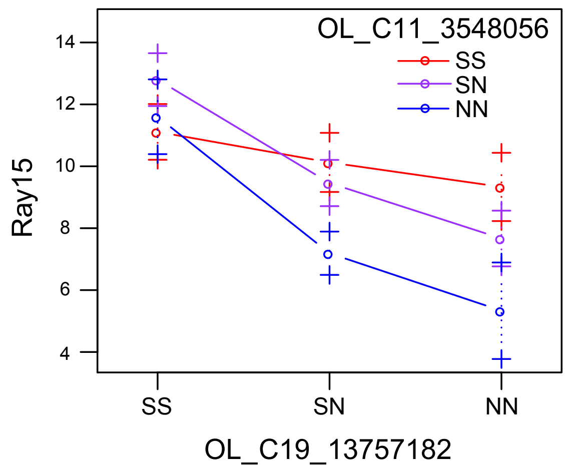

Figure S2 Effects of interaction between LG11 (OL_C11_3548056) and LG19 (OL_C19_13757182) on the papillary process number in the OFAM family.
