# Supplementary material for: Genetic Architecture of the Variation in Male-Specific Ossified Processes on the Anal Fins of Japanese Medaka
Source: G3 (Bethesda). 2015 Oct 26;5(12):2875–84. doi: 10.1534/g3.115.021956 (PMC4683658; doi:10.1534/g3.115.021956)
Supplement: Supporting Information [file supp_g3.115.021956_FigureS3.pdf]

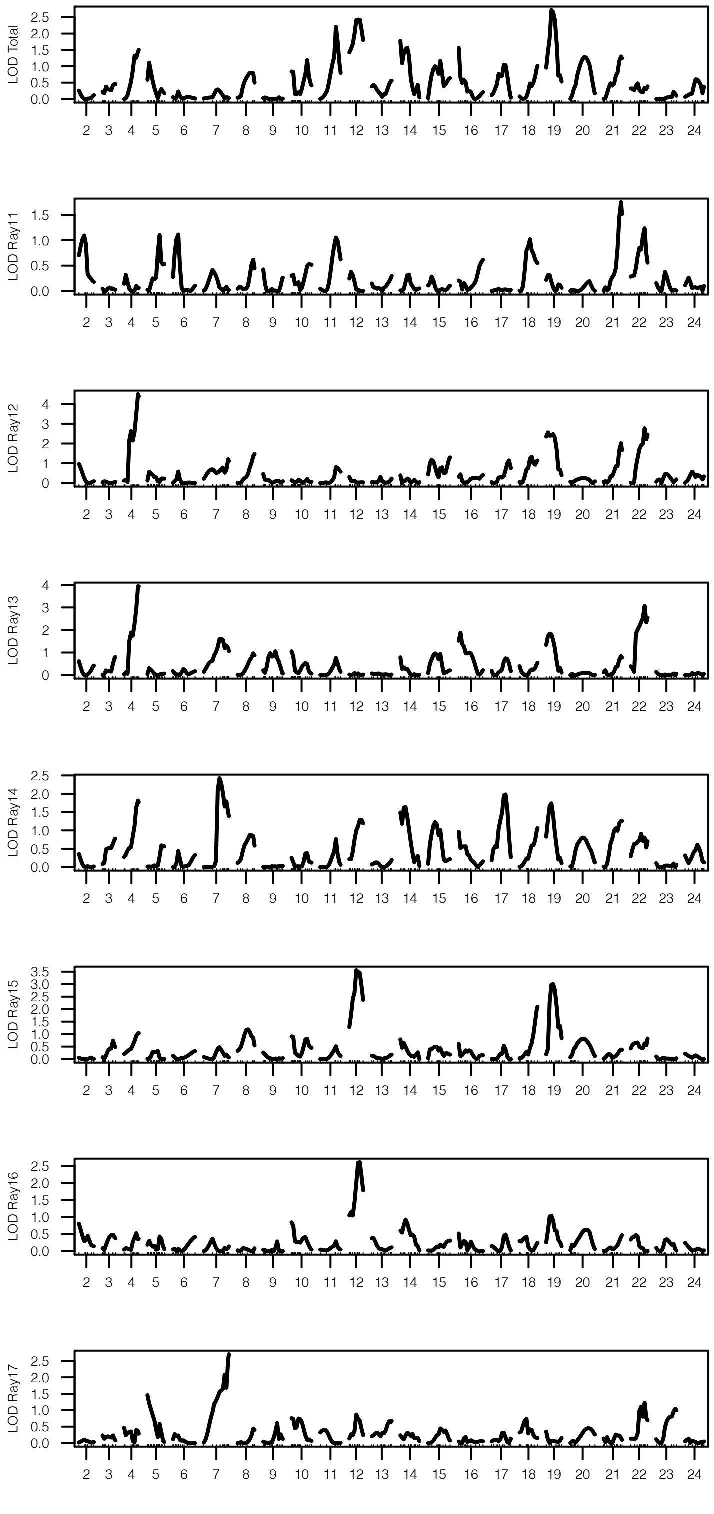

Figure S3 LOD score of the number of total papillary process and the number of papillary process for each fin ray from Ray11 through Ray17 in the AFOM family. The numbers of X-axis shows the number of linkage groups.
