# Supplementary material for: Genetic Architecture of the Variation in Male-Specific Ossified Processes on the Anal Fins of Japanese Medaka
Source: G3 (Bethesda). 2015 Oct 26;5(12):2875–84. doi: 10.1534/g3.115.021956 (PMC4683658; doi:10.1534/g3.115.021956)
Supplement: Supporting Information [file supp_g3.115.021956_FigureS4.pdf]

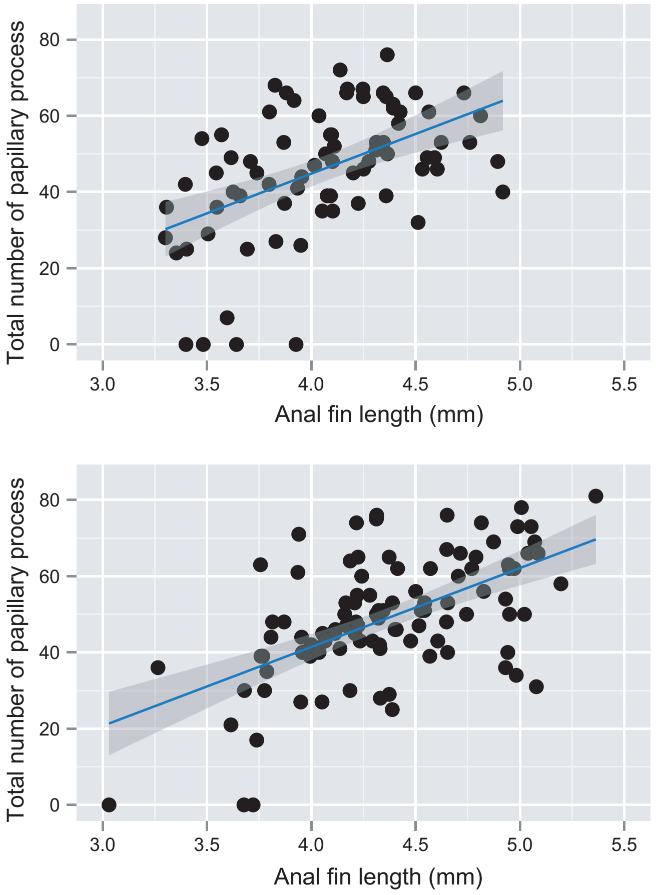

Figure S4 Correlations between the anal fin length and the total number of papillary processes in the OFAM (upper panel) and AFOM families (lower panel). Lines indicate the regression lines, while gray shades indicate 95% confidence intervals.
