# Supplementary material for: Genetic Architecture of the Variation in Male-Specific Ossified Processes on the Anal Fins of Japanese Medaka
Source: G3 (Bethesda). 2015 Oct 26;5(12):2875–84. doi: 10.1534/g3.115.021956 (PMC4683658; doi:10.1534/g3.115.021956)
Supplement: Supporting Information [file supp_g3.115.021956_FigureS5.pdf]

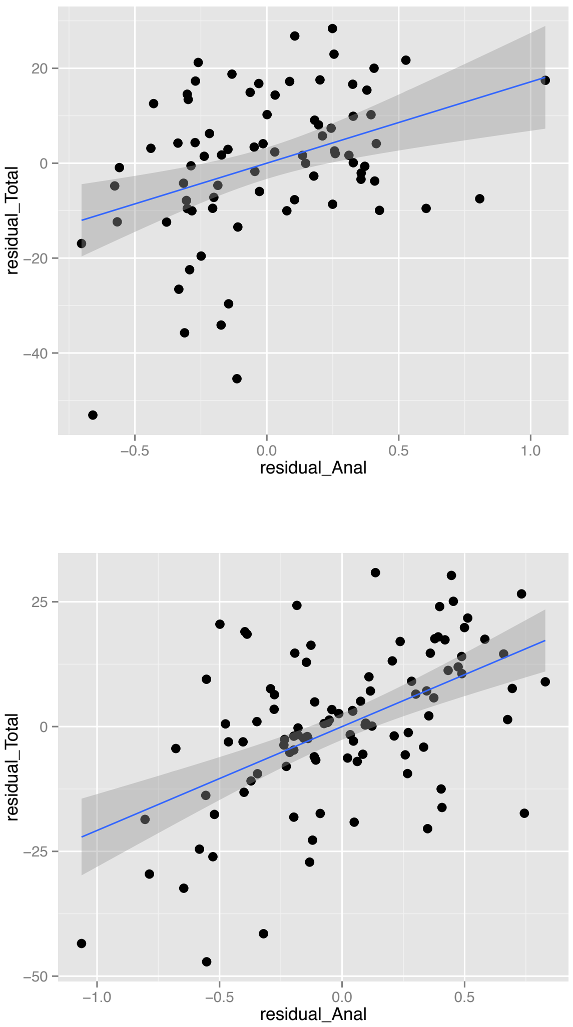

Figure S5 Correlations between the residuals of anal fin length regressed against standard length (X-axis) and the residuals of the total number of papillary processes regressed against standard length (Y-axis) in the OFAM (upper panel) and AFOM families (lower panel). Lines indicate the regression lines, while gray shades indicate 95% confidence intervals.
