# Supplementary material for: Genetic Architecture of the Variation in Male-Specific Ossified Processes on the Anal Fins of Japanese Medaka
Source: G3 (Bethesda). 2015 Oct 26;5(12):2875–84. doi: 10.1534/g3.115.021956 (PMC4683658; doi:10.1534/g3.115.021956)
Supplement: Supporting Information [file supp_g3.115.021956_FigureS6.pdf]

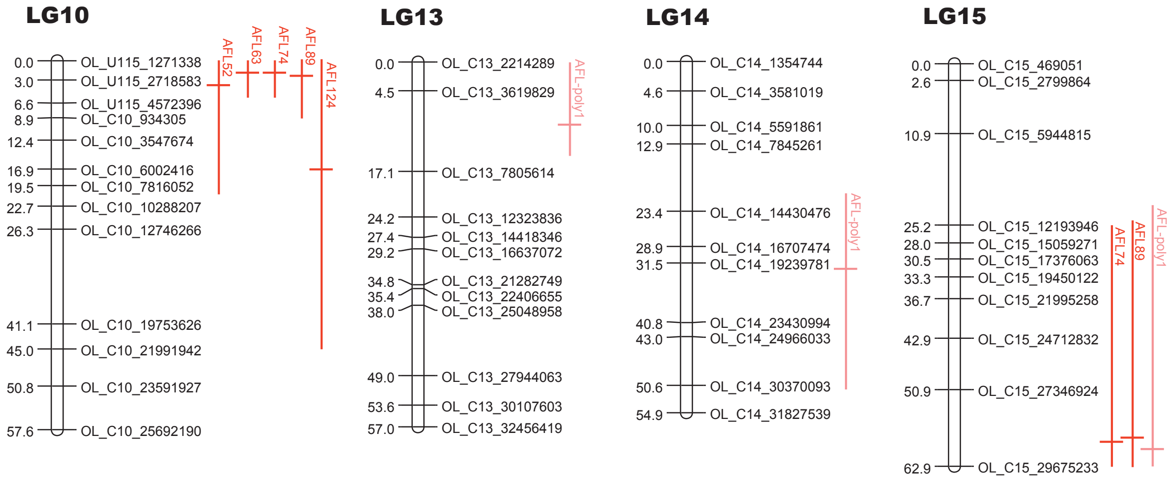

Figure S6 Significant QTL and 95% Bayesian credible intervals mapped on the linkage groups (LG) in AFOM. Only LG that have significant QTL for anal fin length (AFL) are shown here. The letters after the traits indicate the QTL detected at days after fertilization (DAF) or the order of orthogonal polynomials (poly0, poly1, and poly3).
