# Supplementary material for: Genetic Architecture of the Variation in Male-Specific Ossified Processes on the Anal Fins of Japanese Medaka
Source: G3 (Bethesda). 2015 Oct 26;5(12):2875–84. doi: 10.1534/g3.115.021956 (PMC4683658; doi:10.1534/g3.115.021956)
Supplement: Supporting Information [file supp_g3.115.021956_FigureS7.pdf]

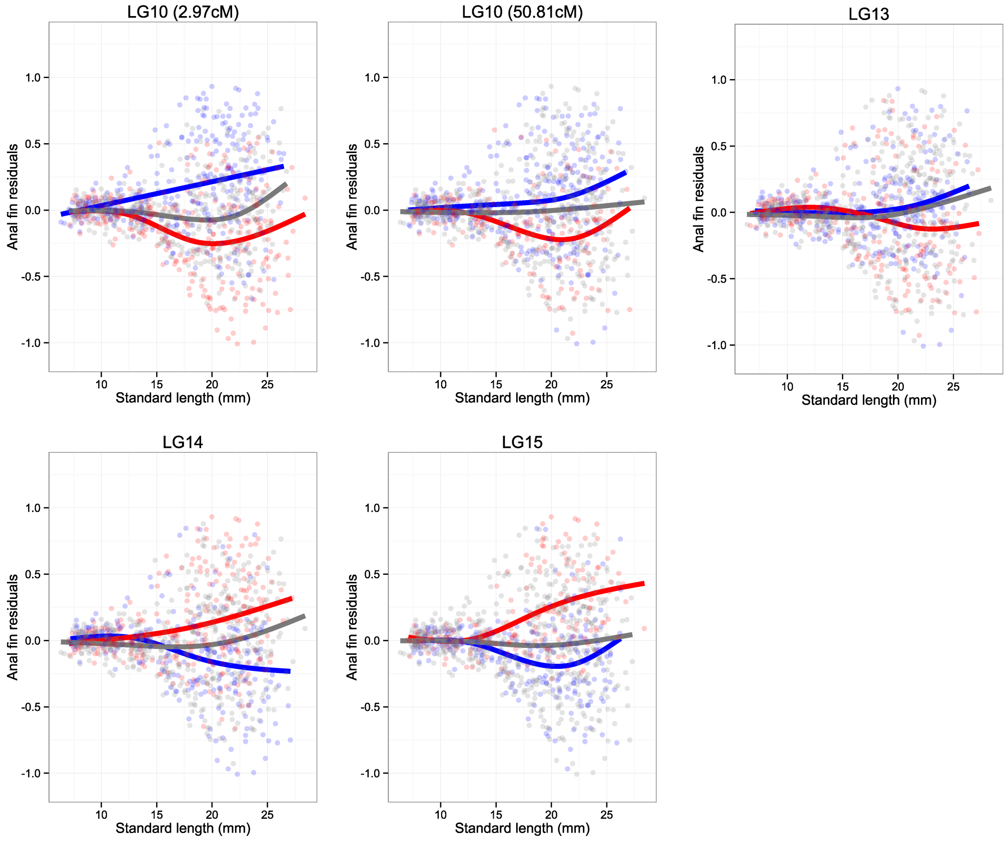

Figure S7 QTL effects on the residuals of fin length in the AFOM shown against standard length; red, homozygote of the southern population alleles; gray, heterozygote; blue, homozygote of the northern population alleles.
