# Supplementary material for: Genetic Architecture of the Variation in Male-Specific Ossified Processes on the Anal Fins of Japanese Medaka
Source: G3 (Bethesda). 2015 Oct 26;5(12):2875–84. doi: 10.1534/g3.115.021956 (PMC4683658; doi:10.1534/g3.115.021956)
Supplement: Supporting Information [file supp_g3.115.021956_TableS2.pdf]

Table S2 QTLs for the number of papillary processes analyzed with standard length as a covariate in the OFAM family

Trait	LG	Location (cM)	95%BI (cM)	Nearest maker	LOD	*P*-value (genome-wide permutation)
Total	11	42	19.6-57.8	OL_C11_26222561	4.97	0.007
Ray12	22	48	0-56.3	OL_C22_23194172	4.24	0.022
Ray14	19	29	11.8-42.6	OL_C19_10378187	4.15	0.024
Ray15	19	28	0-42.6	OL_C19_10378187	3.96	0.03
