# Supplementary material for: Genetic Architecture of the Variation in Male-Specific Ossified Processes on the Anal Fins of Japanese Medaka
Source: G3 (Bethesda). 2015 Oct 26;5(12):2875–84. doi: 10.1534/g3.115.021956 (PMC4683658; doi:10.1534/g3.115.021956)
Supplement: Supporting Information [file supp_g3.115.021956_TableS3.pdf]

**Table S3 QTLs for the number of papillary processes analyzed with anal fin length as a covariate in the OFAM family**

| Trait | LG | Location (cM) | 95%BI (cM) | Nearest maker | LOD | *P*-value (genome-wide permutation) |
|---|---|---|---|---|---|---|
| Total | 11 | 42 | 34.3-57.8 | OL_C11_26222561 | 5.78 | 0.000 |
| Total | 17 | 52.4 | 25.0-58.4 | OL_C17_28352691 | 4.13 | 0.027 |
| Ray12 | 22 | 47 | 17.2-56.3 | OL_C22_20629006 | 5.26 | 0.002 |
| Ray13 | 22 | 43 | 29.0-56.3 | OL_C22_17802287 | 4.38 | 0.016 |
| Ray15 | 11 | 41.0 | 2.1-57.8 | OL_C11_26222561 | 3.86 | 0.030 |
| Ray15 | 19 | 36.8 | 11.8-42.6 | OL_C19_13757182 | 4.36 | 0.009 |
| Ray16 | 11 | 40 | 34.3-57.8 | OL_C11_12552302 | 4.25 | 0.013 |
| Ray16 | 17 | 42 | 13.7-52.4 | OL_C17_23919420 | 5.15 | 0.004 |
| Ray17 | 17 | 25 | 13.7-52.4 | OL_C17_14744358 | 4.34 | 0.026 |
