# Supplementary material for: Genetic Architecture of the Variation in Male-Specific Ossified Processes on the Anal Fins of Japanese Medaka
Source: G3 (Bethesda). 2015 Oct 26;5(12):2875–84. doi: 10.1534/g3.115.021956 (PMC4683658; doi:10.1534/g3.115.021956)
Supplement: Supporting Information [file supp_g3.115.021956_TableS4.pdf]

Table S4 Suggestive QTLs controlling papillary process number in the AFOM.

Trait	LG	Location (cM)	95%BI (cM)	LOD[a]	Threshold[b]	PVE	*P*-value[c]	Nearest maker
Ray12	22	40	18.3-48.5	2.77	2.62	10.9	0.003	OL_C22_17802287
Ray13	22	40	18.3-48.5	3.07	2.96	12.6	0.001	OL_C22_17802287
Ray14	7	45	37.3-68.0	2.43	2.13	9.3	0.007	OL_C7_17169026
Ray15	19	20	0-28.0	3.01	2.94	8.1	0.021	OL_C19_5564885

[a] Peak LOD scores were calculated by MQM analysis.

[b] LOD thresholds of $P < 0.1$ calculated by genome-wide permutation tests are shown.

[c] *P*-values of the effects of genotypes were calculated by the *F*-test of the fitqtl function in R/qtl.
