# Supplementary material for: Genetic Architecture of the Variation in Male-Specific Ossified Processes on the Anal Fins of Japanese Medaka
Source: G3 (Bethesda). 2015 Oct 26;5(12):2875–84. doi: 10.1534/g3.115.021956 (PMC4683658; doi:10.1534/g3.115.021956)
Supplement: Supporting Information [file supp_g3.115.021956_TableS5.pdf]

Table S5 Phenotypic correlations between traits in AFOM

	Anal fin length	Papillary process number
Standard length	0.495 (< 0.001)	0.278 (0.005)
Anal fin length		0.571 (< 0.001)
Papillary process number		

Pearson's correlation coefficients are shown, and *P*-values are shown in parenthesis.
