# Supplementary material for: Genetic Architecture of the Variation in Male-Specific Ossified Processes on the Anal Fins of Japanese Medaka
Source: G3 (Bethesda). 2015 Oct 26;5(12):2875–84. doi: 10.1534/g3.115.021956 (PMC4683658; doi:10.1534/g3.115.021956)
Supplement: Supporting Information [file supp_g3.115.021956_TableS6.pdf]

Table S6 QTLs for the number of papillary processes analyzed with standard length as a covariate in the AFOM family

Trait	LG	Location (cM)	95%BI (cM)	Nearest maker	LOD	*P*-value (genome-wide permutation)
Ray12	4	40	20.1-42.1	OL_C4_26068291	3.98	0.04
Ray13	4	41	31.1-42.1	OL_C4_28493922	3.84	0.048
