# Supplementary material for: Genetic Architecture of the Variation in Male-Specific Ossified Processes on the Anal Fins of Japanese Medaka
Source: G3 (Bethesda). 2015 Oct 26;5(12):2875–84. doi: 10.1534/g3.115.021956 (PMC4683658; doi:10.1534/g3.115.021956)
Supplement: Supporting Information [file supp_g3.115.021956_TableS7.pdf]

Table S7 QTLs for the number of papillary processes analyzed with standard length as a covariate in the AFOM family

Trait	LG	Location (cM)	95%BI (cM)	Nearest maker	LOD	*P*-value (genome-wide permutation)
Total	19	15	0-28.0	OL_C19_5564885	3.76	0.045
Ray15	19	12	0-28.0	OL_C19_3232796	3.98	0.024
