# Supplementary material for: Genetic Architecture of the Variation in Male-Specific Ossified Processes on the Anal Fins of Japanese Medaka
Source: G3 (Bethesda). 2015 Oct 26;5(12):2875–84. doi: 10.1534/g3.115.021956 (PMC4683658; doi:10.1534/g3.115.021956)
Supplement: Supporting Information [file supp_g3.115.021956_TableS8.pdf]

Table S8 QTLs controlling anal fin length and growth in the AFOM family. Significant QTL for fin length at different days after fertilization (DAF) and orthogonal polynomial curve shapes are shown. *P*-values are calculated with genome-wide permutation tests of 1000 bootstraps.

Trait	LG	Location (cM)	95%BI (cM)	LOD	*P*-value (genome-wide permutation)	Nearest maker
Length at DAF52	10	4.00	0.00 - 20.00	5.026	0.003	OL_U115_2718583
Length at DAF63	10	2.00	0.00 - 6.00	6.956	< 0.001	OL_U115_2718583
Length at DAF74	10	2.00	0.00 - 6.00	7.207	< 0.001	OL_U115_2718583
Length at DAF74	15	59.00	25.21 - 62.88	3.922	0.03	OL_C15_29675233
Length at DAF89	10	2.97	0.00 - 8.92	5.440	0.001	OL_U115_2718583
Length at DAF89	15	50.88	25.00 - 62.88	3.833	0.047	OL_C15_27346924
Length at DAF124	10	16.90	0.00 - 45.04	3.819	0.034	OL_C10_6002416
Polynomial order 0	10	4.00	0.00 - 19.47	6.960	< 0.001	OL_U115_2718583
Polynomial order 1	10	2.97	0.00 - 17.00	4.963	0.004	OL_U115_2718583
Polynomial order 1	14	32.00	21.00 - 51.00	3.757	0.051	OL_C14_19239781
Polynomial order 1	15	60.00	22.00 - 62.88	4.240	0.017	OL_C15_29675233
Polynomial order 2	13	10.00	0.00 - 15.00	3.730	0.046	OL_C13_3619829
