# Supplementary material for: Genetic Architecture of the Variation in Male-Specific Ossified Processes on the Anal Fins of Japanese Medaka
Source: G3 (Bethesda). 2015 Oct 26;5(12):2875–84. doi: 10.1534/g3.115.021956 (PMC4683658; doi:10.1534/g3.115.021956)
Supplement: Supporting Information [file supp_g3.115.021956_021956SI.pdf]

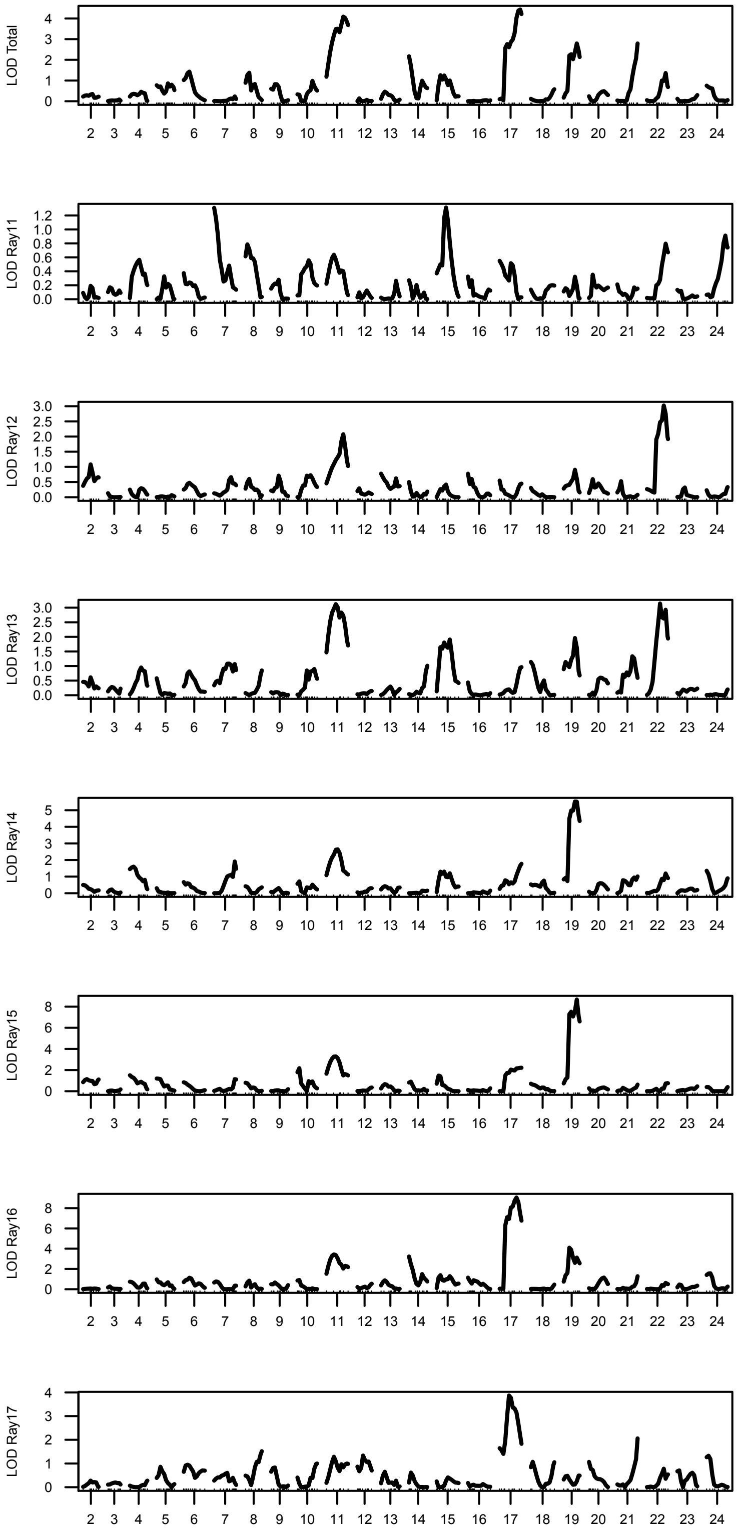

Figure S1 LOD score of the number of total papillary process and the number of papillary process for each fin ray from Ray11 through Ray17 in the OFAM family. The numbers of X-axis shows the number of linkage groups.

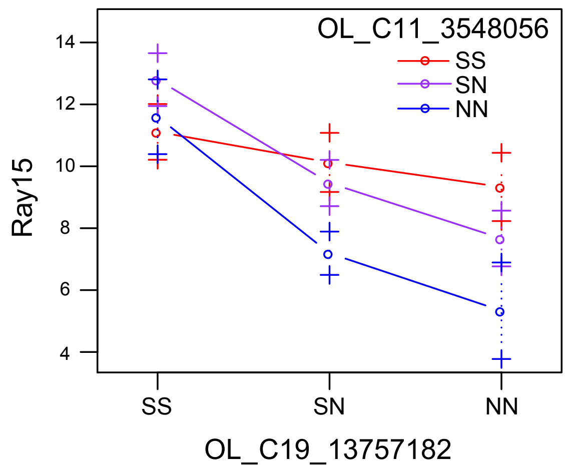

Figure S2 Effects of interaction between LG11 (OL_C11_3548056) and LG19 (OL_C19_13757182) on the papillary process number in the OFAM family.

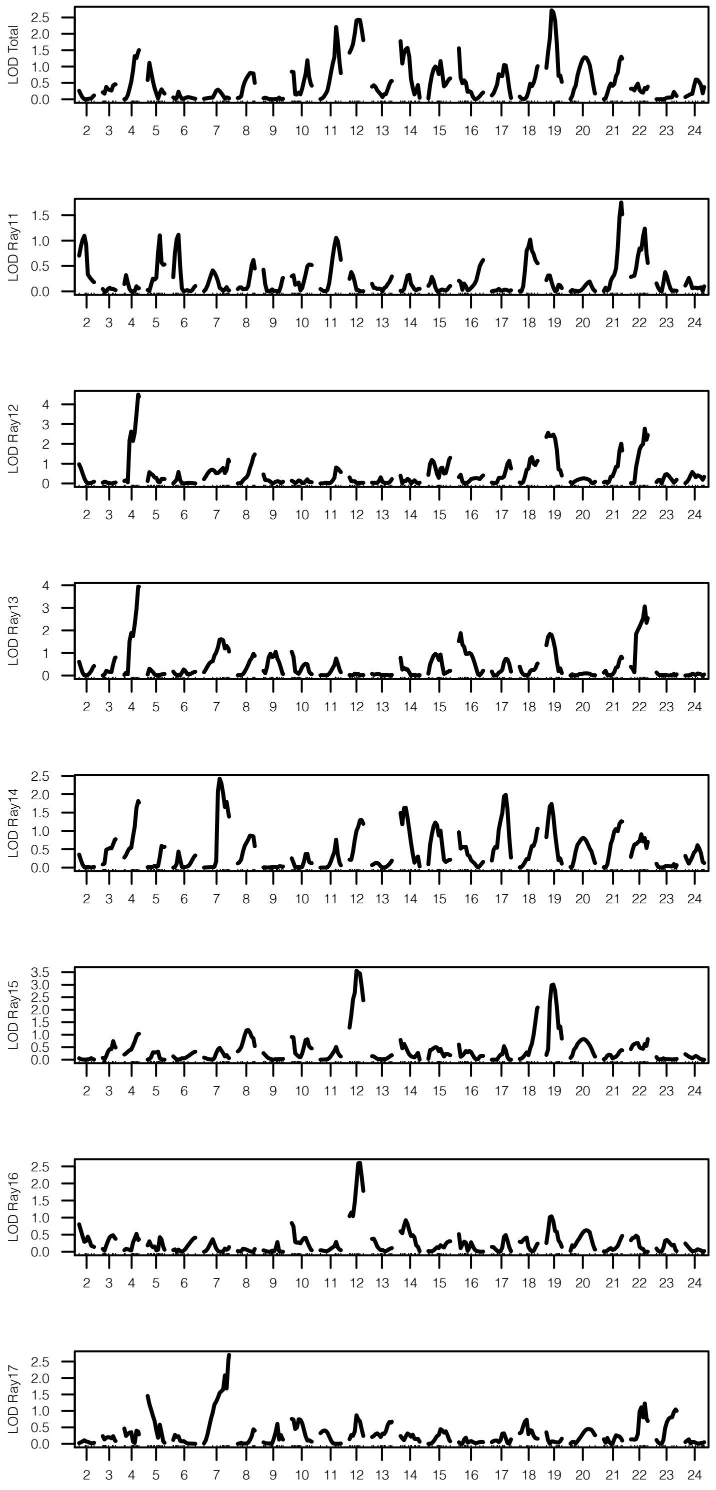

Figure S3 LOD score of the number of total papillary process and the number of papillary process for each fin ray from Ray11 through Ray17 in the AFOM family. The numbers of X-axis shows the number of linkage groups.

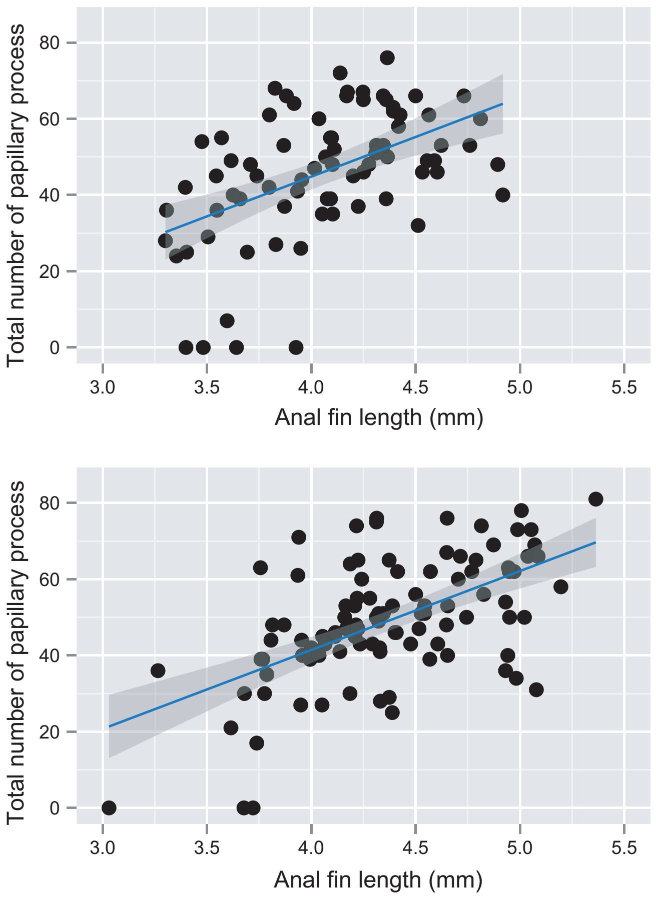

Figure S4 Correlations between the anal fin length and the total number of papillary processes in the OFAM (upper panel) and AFOM families (lower panel). Lines indicate the regression lines, while gray shades indicate 95% confidence intervals.

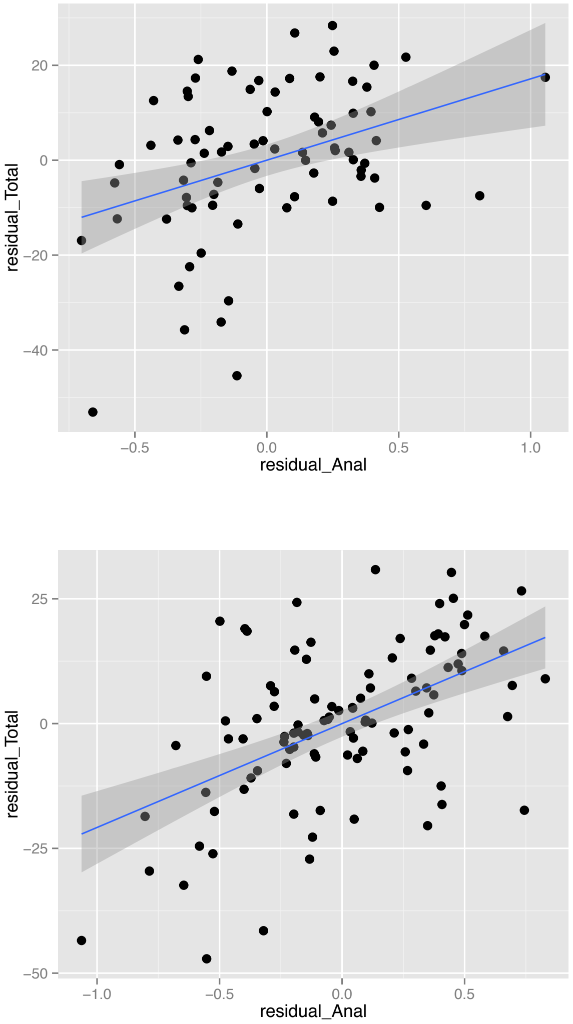

Figure S5 Correlations between the residuals of anal fin length regressed against standard length (X-axis) and the residuals of the total number of papillary processes regressed against standard length (Y-axis) in the OFAM (upper panel) and AFOM families (lower panel). Lines indicate the regression lines, while gray shades indicate 95% confidence intervals.

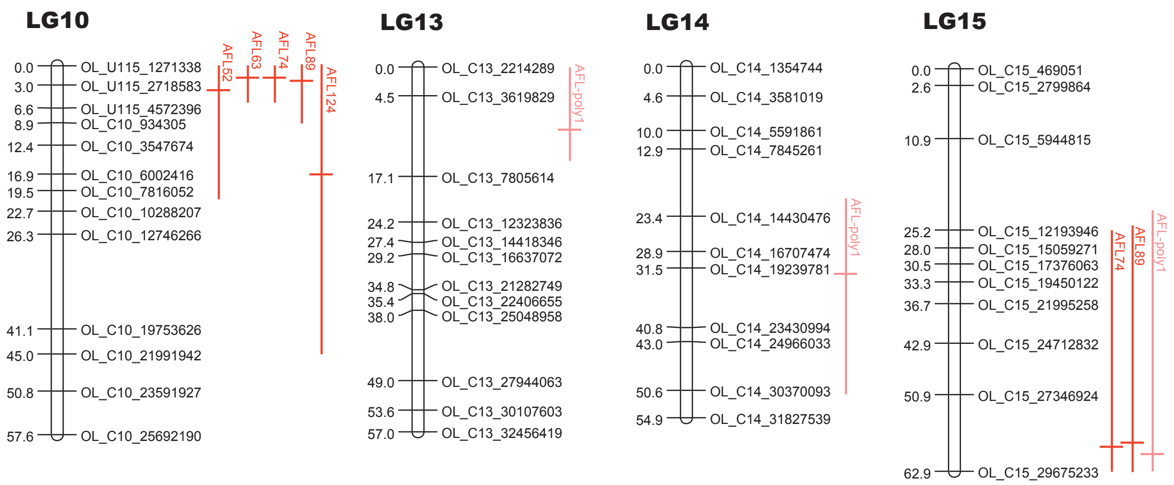

Figure S6 Significant QTL and 95% Bayesian credible intervals mapped on the linkage groups (LG) in AFOM. Only LG that have significant QTL for anal fin length (AFL) are shown here. The letters after the traits indicate the QTL detected at days after fertilization (DAF) or the order of orthogonal polynomials (poly0, poly1, and poly3).

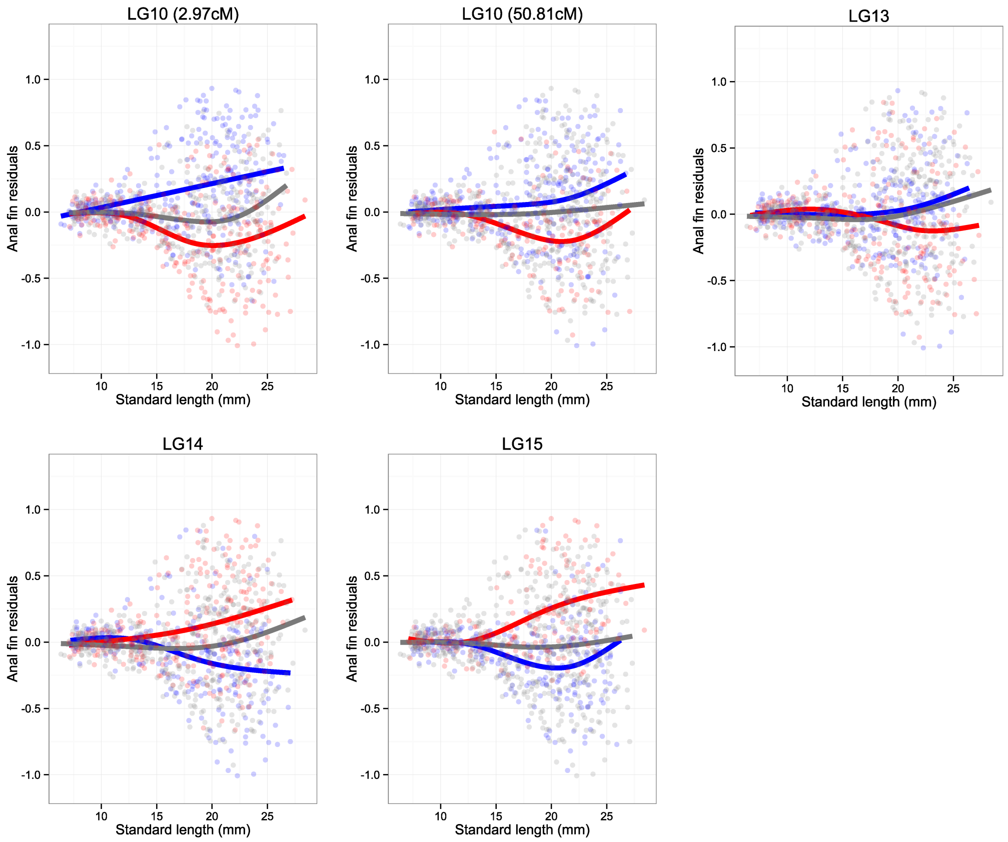

Figure S7 QTL effects on the residuals of fin length in the AFOM shown against standard length; red, homozygote of the southern population alleles; gray, heterozygote; blue, homozygote of the northern population alleles.

Table S1 Phenotypic correlations between traits in the OFAM family

	Anal fin length	Papillary process number
Standard length	0.549 (< 0.001)	0.386 (< 0.001)
Anal fin length		0.499 (< 0.001)
Papillary process number		

Pearson's correlation coefficients are shown, and *P*-values are shown in parenthesis.

Table S2 QTLs for the number of papillary processes analyzed with standard length as a covariate in the OFAM family

Trait	LG	Location (cM)	95%BI (cM)	Nearest maker	LOD	*P*-value (genome-wide permutation)
Total	11	42	19.6-57.8	OL_C11_26222561	4.97	0.007
Ray12	22	48	0-56.3	OL_C22_23194172	4.24	0.022
Ray14	19	29	11.8-42.6	OL_C19_10378187	4.15	0.024
Ray15	19	28	0-42.6	OL_C19_10378187	3.96	0.03

Table S3 QTLs for the number of papillary processes analyzed with anal fin length as a covariate in the OFAM family

Trait	LG	Location (cM)	95%BI (cM)	Nearest maker	LOD	*P*-value (genome-wide permutation)
Total	11	42	34.3-57.8	OL_C11_26222561	5.78	0.000
Total	17	52.4	25.0-58.4	OL_C17_28352691	4.13	0.027
Ray12	22	47	17.2-56.3	OL_C22_20629006	5.26	0.002
Ray13	22	43	29.0-56.3	OL_C22_17802287	4.38	0.016
Ray15	11	41.0	2.1-57.8	OL_C11_26222561	3.86	0.030
Ray15	19	36.8	11.8-42.6	OL_C19_13757182	4.36	0.009
Ray16	11	40	34.3-57.8	OL_C11_12552302	4.25	0.013
Ray16	17	42	13.7-52.4	OL_C17_23919420	5.15	0.004
Ray17	17	25	13.7-52.4	OL_C17_14744358	4.34	0.026

Table S4 Suggestive QTLs controlling papillary process number in the AFOM.

Trait	LG	Location (cM)	95%BI (cM)	LOD[a]	Threshold[b]	PVE	*P*-value[c]	Nearest maker
Ray12	22	40	18.3-48.5	2.77	2.62	10.9	0.003	OL_C22_17802287
Ray13	22	40	18.3-48.5	3.07	2.96	12.6	0.001	OL_C22_17802287
Ray14	7	45	37.3-68.0	2.43	2.13	9.3	0.007	OL_C7_17169026
Ray15	19	20	0-28.0	3.01	2.94	8.1	0.021	OL_C19_5564885

[a] Peak LOD scores were calculated by MQM analysis.

[b] LOD thresholds of $P < 0.1$ calculated by genome-wide permutation tests are shown.

[c] *P*-values of the effects of genotypes were calculated by the *F*-test of the fitqtl function in R/qtl.

Table S5 Phenotypic correlations between traits in AFOM

	Anal fin length	Papillary process number
Standard length	0.495 (< 0.001)	0.278 (0.005)
Anal fin length		0.571 (< 0.001)
Papillary process number		

Pearson's correlation coefficients are shown, and *P*-values are shown in parenthesis.

Table S6 QTLs for the number of papillary processes analyzed with standard length as a covariate in the AFOM family

Trait	LG	Location (cM)	95%BI (cM)	Nearest maker	LOD	*P*-value (genome-wide permutation)
Ray12	4	40	20.1-42.1	OL_C4_26068291	3.98	0.04
Ray13	4	41	31.1-42.1	OL_C4_28493922	3.84	0.048

Table S7 QTLs for the number of papillary processes analyzed with standard length as a covariate in the AFOM family

Trait	LG	Location (cM)	95%BI (cM)	Nearest maker	LOD	*P*-value (genome-wide permutation)
Total	19	15	0-28.0	OL_C19_5564885	3.76	0.045
Ray15	19	12	0-28.0	OL_C19_3232796	3.98	0.024

Table S8 QTLs controlling anal fin length and growth in the AFOM family. Significant QTL for fin length at different days after fertilization (DAF) and orthogonal polynomial curve shapes are shown. *P*-values are calculated with genome-wide permutation tests of 1000 bootstraps.

Trait	LG	Location (cM)	95%BI (cM)	LOD	*P*-value (genome-wide permutation)	Nearest maker
Length at DAF52	10	4.00	0.00 - 20.00	5.026	0.003	OL_U115_2718583
Length at DAF63	10	2.00	0.00 - 6.00	6.956	< 0.001	OL_U115_2718583
Length at DAF74	10	2.00	0.00 - 6.00	7.207	< 0.001	OL_U115_2718583
Length at DAF74	15	59.00	25.21 - 62.88	3.922	0.03	OL_C15_29675233
Length at DAF89	10	2.97	0.00 - 8.92	5.440	0.001	OL_U115_2718583
Length at DAF89	15	50.88	25.00 - 62.88	3.833	0.047	OL_C15_27346924
Length at DAF124	10	16.90	0.00 - 45.04	3.819	0.034	OL_C10_6002416
Polynomial order 0	10	4.00	0.00 - 19.47	6.960	< 0.001	OL_U115_2718583
Polynomial order 1	10	2.97	0.00 - 17.00	4.963	0.004	OL_U115_2718583
Polynomial order 1	14	32.00	21.00 - 51.00	3.757	0.051	OL_C14_19239781
Polynomial order 1	15	60.00	22.00 - 62.88	4.240	0.017	OL_C15_29675233
Polynomial order 2	13	10.00	0.00 - 15.00	3.730	0.046	OL_C13_3619829
